# A Novel Subfoveal Perfluorocarbon Liquid Removal Technique Combining a 25-Gauge Retrobulbar Needle With a Built-in 30-Gauge Needle

**DOI:** 10.3389/fmed.2022.894991

**Published:** 2022-05-31

**Authors:** Yuan Yang, Haodong Xiao, Xuerui Zhang, Wei Mi, Xiaohan Wang, Hongfei Ye, Yanjun Wen, Jie Peng, Peiquan Zhao

**Affiliations:** Department of Ophthalmology, Xinhua Hospital Affiliated to Shanghai Jiao Tong University School of Medicine, Shanghai, China

**Keywords:** subfoveal, perfluorocarbon liquid, 30-gauge needle, retinotomy, retinal detachment

## Abstract

**Introduction:**

To report a novel combining a 25-gauge retrobulbar needle with a built-in 30-gauge needle surgical technique for subfoveal perfluorocarbon liquid (PFCL) removal.

**Materials and Methods:**

Fourteen eyes of 14 patients who underwent subfoveal PFCL removal with a 25-gauge retrobulbar needle combined with a built-in 30-gauge needle were studied. The 30-gauge needle was inserted into the 25-gauge retrobulbar needle. The bent tip of the built-in 30-gauge needle was used to create a 30-gauge retinotomy at the farthest edge of the subfoveal PFCL droplet. Then, a flute cannula was used to aspirate the PFCL through the previously created retinotomy. The best-corrected visual acuity (BCVA) was determined, previous surgical history and post-operative complications were recorded.

**Results:**

Fourteen cases were analyzed. Most eyes (92.85%) showed an improvement in BCVA after surgery. The mean change in the BCVA was −0.7 ± 0.72 logarithm of the minimum angle of resolution (logMAR) units (*p* = 0.006). Post-operative complications included a self-healing macular hole in one eye and vitreous hemorrhage in one eye. Post-operative optical coherence tomography confirmed removal of the subfoveal PFCL with restoration of the macular fovea.

**Conclusion:**

Combining a 25-gauge retrobulbar needle with a built-in 30-gauge needle to remove subfoveal PFCL is easy to perform and carries little potential risk of subretinal impairment. This method also provides relatively good macular contour with functional improvement.

## Introduction

The application of perfluorocarbon liquid (PFCL) in intraocular surgery is well established (1). Its unique physical properties, such as optical clarity and transparency, high specific gravity, moderate surface tension and low viscosity, make it an ideal intraoperative tool for managing proliferative vitreoretinopathy, giant retinal tears or other complicated conditions (2, 3). PFCL can be used to flatten a detached retina, displace subretinal fluid anteriorly and provide countertraction during various surgical maneuvers (4). Inadvertent migration of PFCL into the subretinal space, especially the subfoveal space, is a known complication of using PFCL, occurring in approximately 1 to 11% of vitreoretinal surgeries (5, 6). Rigid retina combined with posterior retinal defects and injecting PFCL with a short needle instead of dual bore cannula are the most common causes of subfoveal PFCL. Other surgical factors associated with subfoveal PFCL retention were the presence of a large peripheral retinotomy, especially if it was 360°, and insufficient saline rinsing during fluid-air exchange (4).

Although many adverse effects associated with subretinal PFCL retention have been reported, the existence of such tiny droplets will only be of concern for symptomatic patients. Extrafoveal PFCL usually does not migrate into the subfoveal space, nor does it cause significant complications. The long-term existence of subfoveal PFCL droplets may constitute a risk of retinal degeneration, gravity deformation, barrier effects, retinal pigment epithelium (RPE) and photoreceptor toxicity, which can induce irreversible glial proliferation, retinal damage and vision loss (7, 8). The timely detection and removal of subfoveal PFCL are conducive to restoring vision (8). Several techniques for subfoveal PFCL removal have been reported, including direct aspiration with various small-gauge cannulas and needles, as well as displacement of the PFCL either through retinotomy or peripherally, with or without subsequent removal (3). Here, we report a reliable surgical procedure combining a 25-gauge retrobulbar needle with a built-in 30-gauge needle to remove subfoveal PFCL. This is a safe, effective and easy-to-perform technique that requires no special instruments. In addition, it provides relatively good macular contour with functional improvement.

## Materials and Methods

Fourteen eyes of 14 patients who underwent subfoveal PFCL removal with a 25-gauge retrobulbar combined with a built-in 30-gauge needle between December 2017 and June 2021 were retrospectively evaluated. All surgeries were performed by the same experienced surgeon (PZ) at Xinhua Hospital Affiliated to Shanghai Jiao Tong University School of Medicine. This study adhered to the tenets of the Declaration of Helsinki. Ethics Committee approval was obtained from the Xinhua Hospital review board. Informed consent was obtained from all patients or their guardians, and possible complications of the procedure were explained.

Medical records containing information on demographics and previous surgical manipulations were obtained. Standard ophthalmological examination records, such as the pre-operative and final best-corrected visual acuity (BCVA), slit-lamp evaluation findings, intraocular pressure (IOP), follow-up duration, spectral domain optical coherence tomography (SD-OCT) findings (RTVue-100, Optovue, Inc., Fremont, CA, United States), and ultrawide-field scanning laser ophthalmoscopy (UWF-SLO) imaging findings (Optos^®^ PLC, Dunfermline, Scotland, United Kingdom), were also obtained. The BCVA was measured as the total number of letters on the Early Treatment Diabetic Retinopathy Study (ETDRS) visual acuity chart when assessed at a starting distance of 4 m.

For statistical convenience, the BCVA results were converted into logarithm of the minimum angle of resolution (logMAR) units for calculation. The Wilcoxon signed-rank test was used to determine the significance of any association between the pre-operative and post-operative BCVA. A *p*-value less than 0.05 was considered significant. Statistical analyses were performed using SPSS for Mac software (version 25.0, IBM Corp., NY, United States).

## Surgical Technique

**FIGURE 1:**
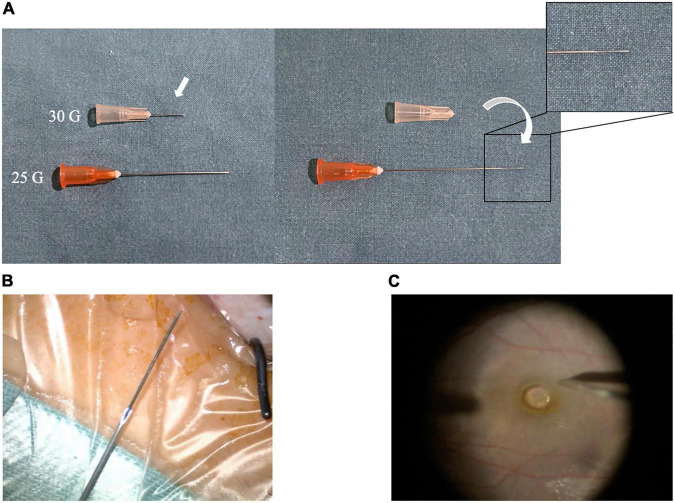
Structure of the combined instrument. **(A)** 25-Gauge retrobulbar needle and 30-Gauge needle. **(B)** Combined 25-gauge retrobulbar needle with built-in 30-gauge needle. **(C)** Intraoperative operation.

After pre-operative preparation and retrobulbar or general anesthesia, a standard 23-gauge pars plana vitrectomy (PPV) was performed. A combined needle consisting of a 25-gauge retrobulbar needle (0.5 mm × 38 mm, TWLB L, KDL^®^, Zhejiang, China) with a built-in 30-gauge needle (0.3 mm × 13 mm, DG34404, BD PrecisionGlide™ Needle, NJ, United States) was used to remove subfoveal PFCL droplets. An ophthalmic needle holder was used to pull out the 30-gauge needle and then insert it into the 25-gauge retrobulbar needle (Figure 1). The outer diameter of the 30-gauge needle is 0.3 mm, and the inner diameter of the 25-gauge needle is 0.32 mm. When the 30-gauge needle was pulled out, the end of the needle was rough. The needle was inserted with labor to make it tight. The friction is sufficient to make the assembled needles connected tightly and prevent the 30-gauge needle from slipping through the 25-gauge needle. The bent tip of the built-in 30-gauge needle was used to create a 30-gauge retinotomy at the farthest edge of the subfoveal PFCL. Then, the flat silicone tip of a flute cannula (Rumex, Inc., FL, United States) was used to aspirate the PFCL through the previously created retinotomy, aspiration was applied under direct visualization to reattach the retina. Fluid-air exchange was performed to assist with retinotomy closure, followed by perfluoropropane (C3F8) gas tamponade. No further treatment for the self-sealing posterior retinotomy was required. Topical steroids were administered to all patients post-operatively. The patients were instructed to maintain a face-down position for at least 3 days. The surgical procedure was recorded by video (Supplementary Video 1).

## Results

Fourteen eyes of 14 patients with subfoveal PFCL were treated by this procedure 1–23 months after the previous vitreoretinal surgery. Eleven patients were male, and 3 patients were female, with a mean age of 30.88 ± 19.66 years and an age range of 11–71 years.

All patients had retinal detachment (RD) as the primary indication for the use of PFCL, including 5 cases of traumatic RD, 4 cases of recurrent RD, 1 case of exudative RD (ERD) caused by retinitis pigmentosa (RP), 1 case of hemorrhagic RD secondary to polypoidal choroidal vasculopathy (PCV) with massive subretinal hemorrhage, 1 case of tractional RD (TRD) caused by retinal capillary hemangioma (RCH), 1 case of combined ERD and TRD caused by familial exudative vitreoretinopathy (FEVR) and 1 case of TRD that lasted for 8 years after congenital cataract surgery. Since nearly all patients had complicated primary vitreoretinopathies, 9 of them had a history of multiple surgeries for RD, and the number of previous operations varied from 1 to 8. Only one patient mentioned central scotoma, which significantly improved after the operation. All other patients were asymptomatic, and retained subfoveal PFCL droplets were not detected until the first post-operative follow-up visit. The mean post-operative follow-up period was 17.43 ± 11.86 months, ranging from 6 to 48 months.

When comparing the last follow-up to pre-operative visual acuity, the BCVA was better in 13 eyes (92.85%) and worse in 1 eye (7.15%). The mean pre-operative BCVA was 1.62 ± 0.58 logMAR units, which improved to 0.92 ± 0.51 logMAR units after subfoveal PFCL removal at the last follow-up visit post-operatively (*p* = 0.006). The mean change in the BCVA was −0.7 ± 0.72 logMAR units. In case 13, the patient had RD for 8 years after congenital cataract surgery. He underwent 8 operations before the subfoveal PFCL removal surgery because of recurrent RD. However, vitreous hemorrhage (VH) was found at his 1-month post-operative follow-up, and he underwent another PPV with silicone oil tamponade. Based on his underlying pathology and rapid progression, he had unsatisfactory visual outcomes.

**FIGURE 2:**
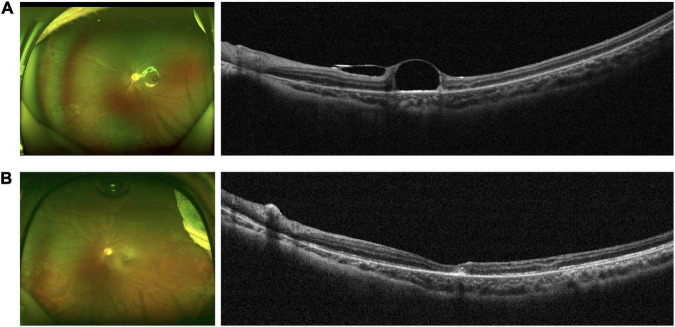
Clinical characteristics of a representative patient (case 5). **(A)** Pre-operative fundus photograph (left) and OCT image (right) showing a subfoveal PFCL droplet. **(B)** Fundus photograph (left) and OCT image (right) confirming removal of the subfoveal PFCL with relatively good macular contour.

Post-operative complications included a self-healing macular hole (MH) in 1 eye (case 7) and VH in 1 eye (case 13). No other intraoperative or post-operative complications (e.g., endophthalmitis, recurrent RD, hypotony, ocular hypertension, submacular hemorrhage, proliferation or fibrosis) related to this PFCL removal technique were noted. Post-operative OCT confirmed removal of the subfoveal PFCL droplets a few days after surgery (Figure 2). Table 1 shows the pre-operative and post-operative clinical characteristics of the patients.

**TABLE 1:** Clinical characteristics of patients undergoing subfoveal PFCL removal surgery.

Case	Sex	Age(Y)	Eye	Intial surgical indications	No. of previous ophthalmic surgeries	Treatment	Lens Status		BCVA(logMAR)		IOP(mmHg)		Follow-up (Months)	Complications
									
							Preop	Final Postop	Preop	Final Postop	Preop	Final Postop		
1	M	30	R	Recurrent RD	2	PPV + PFCL/R + C3F8	Phakic	Phakic	0.92	0.82	14	10	48	—
2	M	71	L	Recurrent RD	2	PPV + SO/R + PFCL/R + C3F8	Pseudophakic	Pseudophakic	1.10	0.82	17	13	14	—
3	F	34	R	Trauma/RD	2	phaco + IOL + PPV + PFCL/R + C3F8	Phakic	Pseudophakic	1.10	0.52	15	14	24	—
4	M	21	L	Recurrent RD	2	PPV + SO/R + PFCL/R + C3F8	Phakic	Phakic	2.00	0.82	14	11	9	—
5	M	11	L	Trauma/RD	1	PPV + SO/R + PFCL/R + C3F8	Phakic	Phakic	2.00	0.70	13	18	9	—
6	M	63	L	Trauma/RD	2	PPV + SO/R + PFCL/R + C3F8	Aphakic	Pseudophakic	1.40	1.22	16	15	7	—
7	F	18	R	Coats’ like RP/ERD	3	phaco + IOL + PPV + PFCL/R + C3F8	Phakic	Pseudophakic	3.00	1.70	17	20	26	MH (Self-Healing)
8	M	53	L	PCV/VH/SMH/RD	1	PPV + SO/R + PFCL/R + C3F8	Aphakic	Pseudophakic	2.00	1.40	11	13	9	—
9	M	31	R	RCH/RD	1	PPV + SO/R + PFCL/R + C3F8	Phakic	Phakic	2.00	0.00	11	17	6	—
10	M	20	L	Recurrent RD	2	PPV + SO/R + PFCL/R + C3F8	Phakic	Phakic	1.10	0.52	37	12	8	—
11	M	12	R	FEVR/RD	1	PPV + SO/R + PFCL/R + C3F8	Aphakic	Aphakic	1.70	0.70	20	17	15	—
12	F	33	L	Trauma/RD	1	PPV + SO/R + PFCL/R + C3F8	Phakic	Phakic	1.40	0.82	13	18	20	—
13	M	14	R	CC/RD	8	PPV + SO/R + PFCL/R + C3F8	Aphakic	Aphakic	1.00	2.00	12	6	32	VH
14	M	25	L	Trauma/RD	5	phaco + IOL + PPV + SO/R + PFCL/R + C3F8	Phakic	Pseudophakic	2.00	0.82	17	18	17	—

*M, male; F, female; R, right; L, left; RD, retinal detachment; RP, retinitis pigmentosa; ERD, exudative retinal detachment; PCV, polypoidal choroidal vasculopathy; VH, vitreous hemorrhage; SMH, submacular hemorrhage; RCH, retinal capillary hemangioma; FEVR, familial exudative vitreoretinopathy; CC, congenital cataract; PPV, pars plana vitrectomy; PFCL/R, perfluorocarbon liquid removal; SO/R, silicone oil removal; IOL, intraocular lens; BCVA, best-corrected visual acuity; IOP, intraocular pressure.*

## Discussion

Perfluorocarbon liquid is commonly used in complicated intraocular surgeries to improve the surgical management of vitreoretinal diseases (9). Spontaneous migration of the PFCL into the subfoveal space through retinal breaks has been described as a rare but prominent complication (8). Subfoveal PFCL droplets always remain stable in size and position, without visible changes for a long period of time (3). Facedown and faceup positions usually cannot displace PFCL droplets away from the fovea. Garcia-Valenzuela et al. analyzed factors that may be associated with subfoveal PFCL retention. According to their retrospective study, related risk factors include a large peripheral retinotomy and the lack of saline irrigation during fluid-air exchange (3, 4, 10).

The toxicity of subfoveal PFCL has been thoroughly studied both in animal experimental models and in humans (8). PFCL droplets impair the delicate interaction between photoreceptors and the RPE, obstruct the delivery of nutrients from the RPE to photoreceptors and weaken the removal of shed photoreceptor segments through RPE phagocytosis (7, 11). Persistence of subfoveal PFCL can induce retinal degeneration and vision-threatening outcomes (8). Therefore, it is important to timely identify and remove subfoveal PFCL, although only a few patients are symptomatic (3).

Different surgical techniques to remove subfoveal PFCLs have been reported, and these techniques can be broadly classified based on either direct aspiration or indirect displacement. One approach is direct aspiration with a small-gauge cannula or needle, ranging from 25 to 50 G, (12–14) with removal of the retained PFCL either through retinotomy or peripherally (3). Internal limiting membrane (ILM) peeling may be performed in previous techniques to help increase the elasticity of the retina and facilitate closure of the retinotomy (15). In this study, we created a retinotomy through the bent tip of the 30-gauge needle, which is a self-sealing retinotomy with little risk of subretinal hemorrhage and retinal injury. Previous experiments have shown that there was no risk of RD after a small, such as 30 gauge retinotomy (16). ILM peeling was not required in our technique, which also simplified the surgical procedure and decreased the possibility of intraoperative mechanical injury. In addition, these disposable syringe needles are commonly used in vitreoretinal surgery and are much less expensive than microcannulas, which allowed us to perform PFCL aspiration surgery with the greatest convenience and at least costs.

Another approach is indirect displacement with or without removal of the PFCL. One method is to create a localized macular RD outside the fovea and gently perform subretinal infusion. However, direct flushing in the subretinal space may lead to damage to the RPE or photoreceptor cells, especially when saline solution is rapidly and forcefully injected. Full-thickness MH formation may be another potential operative complication of this procedure, and areas of retinal thinning overlying the PFCL droplets may rupture secondary to an unstable injection pressure, which is experience dependent (10, 17). Turbulent flow is also associated with retinotomy enlargement and subretinal hemorrhage or fibrosis, resulting in irreversible visual function impairment. Therefore, we chose direct PFCL aspiration through a retinotomy to avoid this subretinal flushing process and to minimize possible retinal damage.

Another method is to create a site of temporary RD at the posterior pole and a retinotomy near the inferotemporal vessels, followed by fluid-air exchange. After these procedures, the patient is asked to maintain an upright position for a short period post-operatively to allow displacement of the PFCL droplets (13). However, PFCL displacement will not eliminate the long-term risk of reverse migration of the PFCL to the macular foveal center, potential retinal toxicity or extramacular retinal hole formation.

Sørensen et al. found that large expansion of the subretinal space and repeated subretinal injection of fluid can damage the RPE, while retinotomy and limited RPE damage are well tolerated. Surgical manipulation and instrumentation in the subretinal space involve a risk of mechanical RPE damage and visual function decline (7). Our technique does not require macular detachment, or involve the entry of any surgical instruments into the subretinal space, which minimizes the damage to the RPE in relation to the subretinal procedures.

According to previous studies, eyes with subfoveal PFCL have better visual outcomes after PFCL removal or displacement (3). Although our patients had complicated coexisting ocular conditions, in this study, we found that the final mean visual acuity improved significantly after subfoveal PFCL removal. Our results demonstrate that early surgical intervention for subfoveal PFCL retention is beneficial. The ultimate visual prognosis may be affected by the location, size, and duration of subfoveal PFCL retention (3, 13). It is important to mention that three of our patients underwent phacoemulsification and intraocular lens (IOL) implantation at the same time as subfoveal PFCL removal, and two patients underwent IOL implantation after this operation. Therefore, the improvement in visual acuity was the result of comprehensive treatment, not only subfoveal PFCL removal. Only one patient mentioned central scotoma before the operation because the preoperative visual acuity of other patients was extremely poor. The central scotoma was significantly improved after the operation.

**FIGURE 3:**
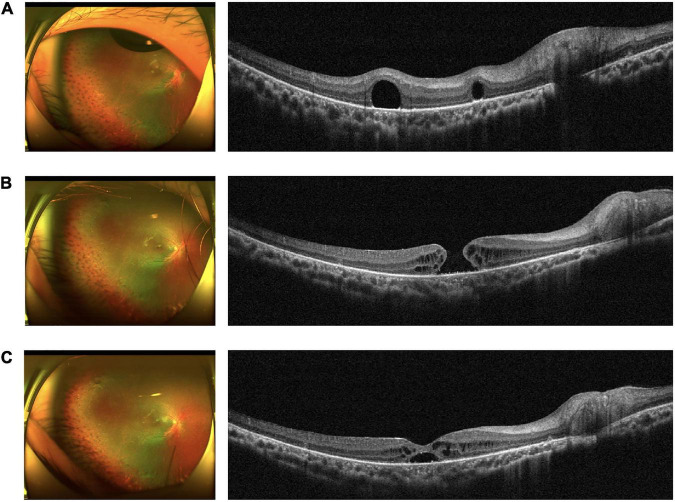
Series of fundus images in case 7, with a self-healing MH as a post-operative complication. **(A)** Pre-operative fundus photograph (left) and OCT image (right) showing a subfoveal PFCL droplet. **(B)** Fundus photograph (left) and OCT image (right) showing a full-thickness MH. **(C)** Fundus photograph (left) and OCT image (right) showing a self-healing MH with macular intraretinal edema and subretinal fluid.

The most common complications of subfoveal PFCL removal surgery were RPE damage, subretinal hemorrhage, proliferation and MH formation (18). The major post-operative complications of our procedure were a self-healing MH in 1 eye (case 7) and VH in 1 eye (case 13). The patient in case 7 was an RP patient with active neovascularization in the retina and ERD in her right eye; she underwent subsequent treatment with two intraocular anti-VEGF drug injections. Subretinal proliferation was found during the follow-up period, and the RD remained, so she underwent PFCL-assisted PPV with C3F8 tamponade. One year after surgery, the patient developed a cataract, and the retina remained attached, so she underwent phacoemulsification and IOL implantation with subfoveal PFCL removal. MH was found after this surgery, which might be related to retinal tissue thinning due to RP. Considering that the patient was unwilling to undergo another operation in a short period of time, we chose to continue observation. Three months later, we were surprised to find that the MH had healed on its own, although there was still subretinal fluid that needed to be absorbed (Figure 3). The patient in case 13 showed VH at his 1-month post-operative follow-up. However, there was no VH or subretinal hemorrhage during the operation, and all his examinations before discharge showed that the retina was attached. Considering that he had undergone 8 operations before this surgery, we considered his VH to be related to his underlying pathology and rapid progression.

There are limitations to our study, including the small sample size, limited follow-up period, and lack of a control group. A longer follow-up period is needed to further assess retinal morphological restoration, visual functional improvement and post-operative complications. Our results show that using a 25-gauge retrobulbar needle combined with a built-in 30-gauge needle to remove subfoveal PFCL seems to be a safe and effective technique with good visual outcomes and few potential complications.

## Conclusion

In conclusion, our technique combining a 25-gauge retrobulbar needle with a built-in 30-gauge needle might be useful for subfoveal PFCL displacement. This technique is easy to perform, achieves relatively good macular contour with functional improvement, and carries little risk of subretinal impairment. However, a longer follow-up observation period is required to examine the long-term anatomical and functional outcomes associated with this technique.

## Data Availability Statement

The raw data supporting the conclusions of this article will be made available by the authors, without undue reservation.

## Ethics Statement

This studies involving human participants were reviewed and approved by the Xinhua Hospital review board. Written informed consent was obtained from the minor(s)’ legal guardian/next of kin for the publication of any potentially identifiable images or data included in this article.

## Author Contributions

PZ and JP: conceptualization and funding acquisition. YY and PZ: methodology. YY, HX, XZ, and WM: data curation and writing – original draft preparation. XW and HY: writing – review and editing. YW: supervision. All authors contributed to the article and approved the submitted version.

## Conflict of Interest

The authors declare that the research was conducted in the absence of any commercial or financial relationships that could be construed as a potential conflict of interest.

## Publisher’s Note

All claims expressed in this article are solely those of the authors and do not necessarily represent those of their affiliated organizations, or those of the publisher, the editors and the reviewers. Any product that may be evaluated in this article, or claim that may be made by its manufacturer, is not guaranteed or endorsed by the publisher.

## Supplementary Material

Supplementary Video 1 Video 1-Surgical procedure of the subfoveal PFCL removal technique.
